# 5-(4-Hy­droxy-3-meth­oxy­benz­yl)-1,3-thia­zolidine-2,4-dione monohydrate

**DOI:** 10.1107/S1600536810049895

**Published:** 2010-12-04

**Authors:** Li-Yan Xiong, Ting-Fang Wang, Li-Ping Zheng, Chuan Zhang, Feng-Chun Wang

**Affiliations:** a New Drug Research Center, School of Pharmacy, Second Military Medical University, Shanghai 200433, People’s Republic of China; b School of Pharmacy, Fujian University of Traditional Chinese Medicine, Fuzhou 350108, People’s Republic of China; c Department of Medicine, The General Hospital of Beijing Military Command, Beijing 100700, People’s Republic of China.

## Abstract

In the title compound, C_11_H_11_NO_4_S·H_2_O, the five-membered thia­zolidine ring is nearly planar, with a maximum deviation of 0.010 (2) Å. The dihedral angle between the thia­zolidine and benzene rings is 49.16 (9)°. Inter­molecular O—H⋯O and N—H⋯O hydrogen bonding is present in the crystal structure.

## Related literature

For the therapeutic and pharmacological properties of thia­zolidinediones, see: Day (1999 ▶); Spiegelman (1998 ▶). For the synthesis of the title compound, see: Madhavan *et al.* (2002 ▶); Shoda *et al.* (1983 ▶). For related structures, see: Divjaković *et al.* (1991 ▶); Yathirajan *et al.* (2005 ▶).
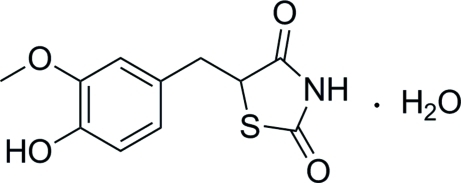


## Experimental

### 

#### Crystal data

C_11_H_11_NO_4_S·H_2_O
*M* *_r_* = 271.28
Monoclinic, 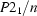

*a* = 10.684 (4) Å
*b* = 8.151 (3) Å
*c* = 14.747 (5) Å
β = 99.657 (4)°
*V* = 1266.0 (8) Å^3^
*Z* = 4
Mo *K*α radiation
μ = 0.27 mm^−1^
*T* = 293 K
0.15 × 0.12 × 0.10 mm

#### Data collection

Bruker SMART 1000 CCD area-detector diffractometer
Absorption correction: multi-scan (*SADABS*; Sheldrick, 1996 ▶) *T*_min_ = 0.960, *T*_max_ = 0.974
4985 measured reflections
2226 independent reflections
1902 reflections with *I* > 2σ(*I*)
*R*_int_ = 0.047

#### Refinement

*R*[*F* ^2^ > 2σ(*F* ^2^)] = 0.043
*wR*(*F* ^2^) = 0.119
*S* = 1.05
2226 reflections
171 parameters
H atoms treated by a mixture of independent and constrained refinement
Δρ_max_ = 0.22 e Å^−3^
Δρ_min_ = −0.31 e Å^−3^

### 

Data collection: *SMART* (Bruker, 2003 ▶); cell refinement: *SAINT* (Bruker, 2003 ▶); data reduction: *SAINT*; program(s) used to solve structure: *SHELXTL* (Sheldrick, 2008 ▶); program(s) used to refine structure: *SHELXTL*; molecular graphics: *SHELXTL*; software used to prepare material for publication: *SHELXTL*.

## Supplementary Material

Crystal structure: contains datablocks I, global. DOI: 10.1107/S1600536810049895/xu5102sup1.cif

Structure factors: contains datablocks I. DOI: 10.1107/S1600536810049895/xu5102Isup2.hkl

Additional supplementary materials:  crystallographic information; 3D view; checkCIF report

## Figures and Tables

**Table 1:** Hydrogen-bond geometry (Å, °)

*D*—H⋯*A*	*D*—H	H⋯*A*	*D*⋯*A*	*D*—H⋯*A*
N3—H3⋯O2^i^	0.86	2.03	2.886 (2)	174
O4—H4*A*⋯O5^ii^	0.82	1.87	2.685 (2)	171
O5—H5*A*⋯O3	0.82 (5)	2.19 (5)	2.962 (2)	156 (4)
O5—H5*A*⋯O4	0.82 (5)	2.37 (4)	2.947 (2)	127 (4)
O5—H5*B*⋯O1^iii^	0.85 (3)	1.97 (3)	2.795 (3)	163 (3)

Symmetry codes: (i) 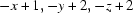; (ii) 
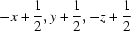
; (iii) 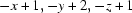.

**Fig. 1.**
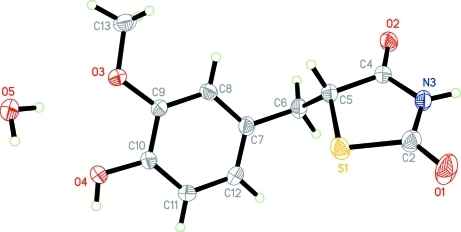
View of the asymmetric unit of the compound showing the atom-labelling scheme. Displacement ellipsoids are drawn at the 30% probability level. H atoms are presented as a small spheres of arbitrary radius.

**Fig. 2.**
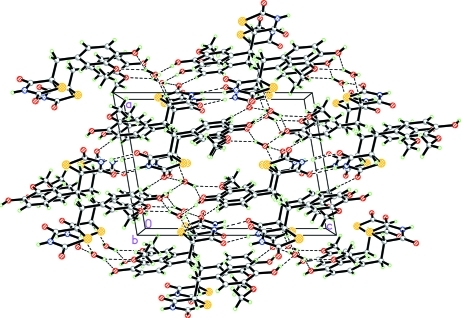
Crystal packing of the title compound. Intermolecular hydrogen bonds are shown as dashed lines.

## supplementary crystallographic information

### Comment

Thiazolidinediones (TZDs), which are known to sensitize tissues to insulin, have been developed and clinically used as antidiabetic agents. They have been shown to reduce plasma glucose, lipid, and insulin levels, and used for the treatment of type 2 diabetes (Day, 1999; Spiegelman, 1998). Prompted by the activity of TZDs, we have synthesized the title compound to study its crystal structure.

The asymmetric unit contains a 5-(4-hydroxy-3-methoxybenzyl)thiazolidine-2,4-dione molecule and a solvate water molecule (Fig. 1). The geometric parameter of the title compoundare to its related structures (Divjakovic *et al.*, 1991; Yathirajan *et al.*, 2005). The dihedral angle between the thiazolidinedione ring [S1/C2/N3/C4/C5] and the benzene ring [C7–C12] is 49.16 (9)°. In the crystal packing (Fig. 2), the molecules are linked *via* intermolecular N1—H1···O2 hydrogen bonds. In addition, the molecule is connected to the water molecule by O5—H5···O1, O5—H5···O4, O5—H5···O3 and O4—H4···O5 hydrogen bonds which generate a three dimensional network (Table 1).

### Experimental

A mixture of 2,4-tThiazolidinedione (3.51 g, 0.03 mol), 4-hydroxy-3-methoxybenzaldehyde (4.56 g, 0.03 mol), acetic acid (0.18 g, 0.003 mol) and piperidine (0.26 g, 0.003 mol) in toluene (60 ml) was refluxed for 5 h with continuous removal of water. The reaction mixture was cooled to room temperature and the resultant crystalline compound was filtered and washed with water and dried to afford the (*Z*)-5-(4-hydroxy-3-methoxybenzylidene)thiazolidine-2,4-dione. Yield=7.33 g, 97.3%. To a solution of (*Z*)-5-(4-hydroxy-3-methoxybenzylidene) thiazolidine-2,4-dione (4 g, 0.016 mol) in 1,4-dioxane (400 ml), hydrogenated in the presence of 10% Pd/C (1.0 g) at 60 psi for 24 h. The mixture was filtered through a bed of Celite. The filtrate was evaporated under reduced pressure and purified by column chromatography using 50:1 CH_2_Cl_2_/MeOH to afford the title compound as yellowish solid. Yield = 1.96 g, 48.6% (Madhavan *et al.*, 2002; Shoda *et al.*, 1983). Crystallization of the product was carried out by dissolving the product in 10 ml a solvent mixture of MeOH and water (4:1) at room temperature.

### Refinement

Water H atoms were located in a difference Fourier map and refined isotropically. Other H atoms were positioned geometrically and refined using the riding-model approximation with C—H = 0.93–0.97, O—H = 0.82 and N—H = 0.86 Å, and U_iso_(H) = 1.5U_eq_(C,O) for methyl H and hydroxy H atoms and 1.2U_eq_(C,N) for the others.

### Crystal data

**Table:**

C_11_H_11_NO_4_S·H_2_O	*F*(000) = 568
*M**_r_* = 271.28	*D*_x_ = 1.423 Mg m^−^^3^
Monoclinic, *P*2_1_/*n*	Mo *K*α radiation, λ = 0.71073 Å
Hall symbol: -P 2yn	Cell parameters from 889 reflections
*a* = 10.684 (4) Å	θ = 2.8–27.5°
*b* = 8.151 (3) Å	µ = 0.27 mm^−^^1^
*c* = 14.747 (5) Å	*T* = 293 K
β = 99.657 (4)°	Block, yellow
*V* = 1266.0 (8) Å^3^	0.15 × 0.12 × 0.10 mm
*Z* = 4	

### Data collection

**Table:**

Bruker SMART 1000 CCD area-detector diffractometer	2226 independent reflections
Radiation source: fine-focus sealed tube	1902 reflections with *I* > 2σ(*I*)
graphite	*R*_int_ = 0.047
Detector resolution: 10.0 pixels mm^-1^	θ_max_ = 25.0°, θ_min_ = 2.2°
φ and ω scans	*h* = −12→12
Absorption correction: multi-scan (*SADABS*; Sheldrick, 1996)	*k* = −9→6
*T*_min_ = 0.960, *T*_max_ = 0.974	*l* = −16→17
4985 measured reflections	

### Refinement

**Table:**

Refinement on *F*^2^	Primary atom site location: structure-invariant direct methods
Least-squares matrix: full	Secondary atom site location: difference Fourier map
*R*[*F*^2^ > 2σ(*F*^2^)] = 0.043	Hydrogen site location: inferred from neighbouring sites
*wR*(*F*^2^) = 0.119	H atoms treated by a mixture of independent and constrained refinement
*S* = 1.05	*w* = 1/[σ^2^(*F*_o_^2^) + (0.0681*P*)^2^ + 0.2943*P*] where *P* = (*F*_o_^2^ + 2*F*_c_^2^)/3
2226 reflections	(Δ/σ)_max_ = 0.001
171 parameters	Δρ_max_ = 0.22 e Å^−^^3^
0 restraints	Δρ_min_ = −0.31 e Å^−^^3^

### Special details

**Table:**

Experimental. 1H NMR (300 MHz, CDCl3): δ 8.72(bar, 1H, N—H), 6.87–6.71 (m, 3H, 8-H, 11-H, 12-H), 5.46 (bar, 1H, 10-OH), 4.47–4.51 (m, 1H, 5-H), 3.83 (s, 3H, 9-OCH3), 3.46 (dd, 1H, j=14.4, 4.2, 3-H), 3.06 (dd, 1H, j=14.1, 9.6, 3-H). 13C NMR (300 MHz, CDCl3): δ 174.5, 167.5, 147.8, 145.7, 132.7, 123.1, 116.7, 114.9, 57.2, 56.1, 36.2. MS(ESI) m/*z* calc. for C11H11NO4S 253.27, found [M–1]+ 252.15. m.p. 109-110°C
Geometry. All e.s.d.'s (except the e.s.d. in the dihedral angle between two l.s. planes) are estimated using the full covariance matrix. The cell e.s.d.'s are taken into account individually in the estimation of e.s.d.'s in distances, angles and torsion angles; correlations between e.s.d.'s in cell parameters are only used when they are defined by crystal symmetry. An approximate (isotropic) treatment of cell e.s.d.'s is used for estimating e.s.d.'s involving l.s. planes.
Refinement. Refinement of *F*^2^ against ALL reflections. The weighted *R*-factor *wR* and goodness of fit *S* are based on *F*^2^, conventional *R*-factors *R* are based on *F*, with *F* set to zero for negative *F*^2^. The threshold expression of *F*^2^ > σ(*F*^2^) is used only for calculating *R*-factors(gt) *etc*. and is not relevant to the choice of reflections for refinement. *R*-factors based on *F*^2^ are statistically about twice as large as those based on *F*, and *R*- factors based on ALL data will be even larger.

### Fractional atomic coordinates and isotropic or equivalent isotropic displacement parameters (Å^2^)

**Table:**

	*x*	*y*	*z*	*U*_iso_*/*U*_eq_	
S1	0.49420 (5)	1.06186 (7)	0.70922 (3)	0.0523 (2)	
N3	0.51317 (16)	1.07172 (19)	0.88568 (11)	0.0430 (4)	
H3	0.5303	1.1072	0.9413	0.052*	
C2	0.5427 (2)	1.1623 (3)	0.81421 (15)	0.0529 (5)	
C4	0.45618 (17)	0.9243 (2)	0.86643 (12)	0.0383 (4)	
C5	0.43481 (18)	0.8857 (2)	0.76418 (12)	0.0403 (4)	
H5	0.4868	0.7902	0.7545	0.048*	
C6	0.29661 (18)	0.8453 (3)	0.72695 (13)	0.0448 (5)	
H6A	0.2706	0.7526	0.7606	0.054*	
H6B	0.2440	0.9383	0.7370	0.054*	
C7	0.27553 (18)	0.8048 (2)	0.62553 (13)	0.0407 (4)	
C8	0.31489 (17)	0.6537 (2)	0.59516 (12)	0.0386 (4)	
H8	0.3523	0.5768	0.6378	0.046*	
C9	0.29866 (16)	0.6178 (2)	0.50237 (12)	0.0360 (4)	
C10	0.24649 (18)	0.7353 (2)	0.43822 (12)	0.0408 (4)	
C11	0.2075 (2)	0.8830 (2)	0.46857 (14)	0.0512 (5)	
H11	0.1718	0.9613	0.4261	0.061*	
C12	0.2206 (2)	0.9167 (2)	0.56175 (14)	0.0504 (5)	
H12	0.1918	1.0163	0.5812	0.061*	
C13	0.3765 (2)	0.3430 (3)	0.52648 (16)	0.0541 (5)	
H13A	0.3955	0.2494	0.4917	0.081*	
H13B	0.3128	0.3141	0.5625	0.081*	
H13C	0.4520	0.3781	0.5665	0.081*	
O1	0.5944 (2)	1.2929 (2)	0.82373 (12)	0.0877 (7)	
O2	0.42480 (14)	0.83322 (17)	0.92352 (9)	0.0483 (4)	
O3	0.33054 (14)	0.47280 (16)	0.46520 (9)	0.0485 (4)	
O4	0.23570 (15)	0.69314 (17)	0.34819 (9)	0.0537 (4)	
H4A	0.2034	0.7691	0.3160	0.080*	
O5	0.36833 (18)	0.4211 (2)	0.27286 (12)	0.0558 (4)	
H5A	0.336 (4)	0.444 (5)	0.318 (3)	0.134 (16)*	
H5B	0.376 (3)	0.518 (4)	0.253 (2)	0.085 (10)*	

### Atomic displacement parameters (Å^2^)

**Table:**

	*U*^11^	*U*^22^	*U*^33^	*U*^12^	*U*^13^	*U*^23^
S1	0.0699 (4)	0.0527 (4)	0.0342 (3)	−0.0171 (2)	0.0079 (2)	0.0023 (2)
N3	0.0578 (10)	0.0384 (9)	0.0326 (8)	−0.0079 (7)	0.0065 (7)	−0.0046 (6)
C2	0.0715 (14)	0.0423 (11)	0.0453 (11)	−0.0124 (10)	0.0112 (10)	−0.0012 (9)
C4	0.0432 (9)	0.0353 (10)	0.0352 (9)	−0.0008 (7)	0.0033 (7)	−0.0011 (7)
C5	0.0510 (10)	0.0365 (10)	0.0332 (9)	−0.0018 (8)	0.0060 (8)	−0.0021 (8)
C6	0.0518 (11)	0.0454 (11)	0.0374 (10)	−0.0063 (9)	0.0079 (8)	−0.0067 (8)
C7	0.0465 (10)	0.0381 (10)	0.0369 (10)	−0.0074 (8)	0.0050 (8)	−0.0055 (8)
C8	0.0441 (9)	0.0368 (10)	0.0337 (9)	−0.0031 (8)	0.0029 (7)	0.0046 (7)
C9	0.0409 (9)	0.0300 (9)	0.0367 (9)	−0.0029 (7)	0.0051 (7)	−0.0018 (7)
C10	0.0509 (10)	0.0363 (10)	0.0328 (9)	−0.0020 (8)	0.0006 (8)	−0.0018 (7)
C11	0.0727 (14)	0.0357 (10)	0.0405 (10)	0.0059 (10)	−0.0042 (9)	0.0021 (8)
C12	0.0679 (13)	0.0344 (10)	0.0466 (11)	0.0016 (9)	0.0028 (10)	−0.0078 (8)
C13	0.0650 (13)	0.0390 (11)	0.0599 (13)	0.0097 (10)	0.0149 (10)	0.0102 (10)
O1	0.1467 (19)	0.0578 (11)	0.0617 (11)	−0.0545 (12)	0.0262 (11)	−0.0083 (8)
O2	0.0667 (9)	0.0429 (7)	0.0340 (7)	−0.0108 (7)	0.0047 (6)	0.0033 (6)
O3	0.0718 (9)	0.0344 (7)	0.0391 (7)	0.0089 (7)	0.0084 (6)	0.0019 (6)
O4	0.0836 (10)	0.0421 (8)	0.0312 (7)	0.0092 (7)	−0.0022 (7)	−0.0009 (6)
O5	0.0797 (11)	0.0438 (9)	0.0440 (8)	0.0040 (8)	0.0106 (8)	−0.0066 (7)

### Geometric parameters (Å, °)

**Table:**

S1—C2	1.751 (2)	C8—H8	0.9300
S1—C5	1.815 (2)	C9—O3	1.370 (2)
N3—C4	1.356 (2)	C9—C10	1.395 (3)
N3—C2	1.366 (3)	C10—O4	1.358 (2)
N3—H3	0.8600	C10—C11	1.373 (3)
C2—O1	1.197 (3)	C11—C12	1.385 (3)
C4—O2	1.211 (2)	C11—H11	0.9300
C4—C5	1.520 (2)	C12—H12	0.9300
C5—C6	1.523 (3)	C13—O3	1.424 (2)
C5—H5	0.9800	C13—H13A	0.9600
C6—C7	1.511 (3)	C13—H13B	0.9600
C6—H6A	0.9700	C13—H13C	0.9600
C6—H6B	0.9700	O4—H4A	0.8200
C7—C12	1.370 (3)	O5—H5A	0.82 (5)
C7—C8	1.399 (3)	O5—H5B	0.85 (3)
C8—C9	1.382 (3)		
			
C2—S1—C5	92.79 (9)	C9—C8—C7	120.60 (17)
C4—N3—C2	118.11 (16)	C9—C8—H8	119.7
C4—N3—H3	120.9	C7—C8—H8	119.7
C2—N3—H3	120.9	O3—C9—C8	125.52 (16)
O1—C2—N3	123.5 (2)	O3—C9—C10	114.77 (15)
O1—C2—S1	125.58 (18)	C8—C9—C10	119.71 (17)
N3—C2—S1	110.93 (15)	O4—C10—C11	124.07 (17)
O2—C4—N3	124.38 (17)	O4—C10—C9	116.61 (16)
O2—C4—C5	123.37 (17)	C11—C10—C9	119.31 (17)
N3—C4—C5	112.25 (16)	C10—C11—C12	120.76 (18)
C4—C5—C6	112.19 (15)	C10—C11—H11	119.6
C4—C5—S1	105.90 (13)	C12—C11—H11	119.6
C6—C5—S1	113.61 (13)	C7—C12—C11	120.64 (19)
C4—C5—H5	108.3	C7—C12—H12	119.7
C6—C5—H5	108.3	C11—C12—H12	119.7
S1—C5—H5	108.3	O3—C13—H13A	109.5
C7—C6—C5	112.25 (15)	O3—C13—H13B	109.5
C7—C6—H6A	109.2	H13A—C13—H13B	109.5
C5—C6—H6A	109.2	O3—C13—H13C	109.5
C7—C6—H6B	109.2	H13A—C13—H13C	109.5
C5—C6—H6B	109.2	H13B—C13—H13C	109.5
H6A—C6—H6B	107.9	C9—O3—C13	118.00 (15)
C12—C7—C8	118.92 (18)	C10—O4—H4A	109.5
C12—C7—C6	120.66 (18)	H5A—O5—H5B	98 (3)
C8—C7—C6	120.40 (17)		

### Hydrogen-bond geometry (Å, °)

**Table:**

*D*—H···*A*	*D*—H	H···*A*	*D*···*A*	*D*—H···*A*
N3—H3···O2^i^	0.86	2.03	2.886 (2)	174.
O4—H4A···O5^ii^	0.82	1.87	2.685 (2)	171.
O5—H5A···O3	0.82 (5)	2.19 (5)	2.962 (2)	156 (4)
O5—H5A···O4	0.82 (5)	2.37 (4)	2.947 (2)	127 (4)
O5—H5B···O1^iii^	0.85 (3)	1.97 (3)	2.795 (3)	163 (3)

Symmetry codes: (i) −*x*+1, −*y*+2, −*z*+2; (ii) −*x*+1/2, *y*+1/2, −*z*+1/2; (iii) −*x*+1, −*y*+2, −*z*+1.
